# Human Interleukin-2 and Hen Egg White Lysozyme: Screening for Bacteriolytic Activity against Various Bacterial Cells

**Published:** 2016

**Authors:** P. A. Levashov, E. D. Ovchinnikova, O. A. Morozova, D. A. Matolygina, H. E. Osipova, T. A. Cherdyntseva, S. S. Savin, G. S. Zakharova, A. A. Alekseeva, N. G. Belogurova, S. A. Smirnov, V. I. Tishkov, A. V. Levashov

**Affiliations:** Department of Chemical Enzymology, Faculty of Chemistry, M.V. Lomonosov Moscow State University, Leninskie Gory, 1-3, 119991, Moscow, Russian Federation; Clinical Center, I.M. Sechenov First Moscow State Medical University, Bolshaya Pirogovskaya St., 2 -4, 119881, Moscow, Russian Federation; Department of Microbiology, Faculty of Biology, M.V. Lomonosov Moscow State University, Leninskie Gory, 1-12, 119991, Moscow, Russian Federation; A.N. Bach Institute of Biochemistry, Federal Research Centre “Fundamentals of Biotechnology” of the Russian Academy of Sciences, Leninsky Pr., 33-2, 119071, Moscow, Russian Federation

**Keywords:** lysozyme, interleukin-2, bacteriolytic activity

## Abstract

The bacteriolytic activity of interleukin-2 and hen egg white lysozyme against 34 different species of microorganisms has been studied. It was found that 6 species of microorganisms are lysed in the presence of interleukin-2. All interleukin-2-sensitive microorganisms belong either to the Enterobacteriaceae, Bacillaceae, or the Lactobacillaceae family. It was also found that 12 species of microorganisms are lysed in the presence of lysozyme, and 16 species of microorganisms are lysed in the presence of sodium dodecyl sulfate (SDS). The bacteriolytic activity of interleukin-2 and lysozyme was studied at various pH values.

## INTRODUCTION

Interleukin-2 (IL-2) is one of the most important regulators of vital activity. This lymphokine is involved in the regulation of such processes as proliferation and differentiation of T lymphocytes, increase of the cytolytic activity of NK cells, proliferation of B lymphocytes, immunoglobulin secretion, etc. We have recently shown that human IL-2 is able to exhibit bacteriolytic activity [1-3]. A comparative test with several bacterial strains has shown that IL-2 has a narrower substrate specificity compared to hen egg white lysozyme. IL-2, as well as lysozyme, is capable of lysing* Escherichia coli *and *Lactobacillus plantarum *cells, but, unlike lysozyme, it shows no effect on *Micrococcus luteus *and *Bacillus subtilis *[1-3]. The detection of IL-2 activity against *E. coli *and *L. plantarum *turned out to be surprising. The mechanism of the bacteriolytic action of IL-2 still remains unknown, and its elucidation requires a study of the influence of IL-2 on other bacterial species. Since IL-2 plays an important role in the development of the immune response and is used as a drug, it is of primal importance to examine its action on the bacteria that are often in contact with humans, including the components of symbiotic microflora.

The main objective of the study was to screen IL-2 for bacteriolytic activity against microorganisms that are found on human skin and mucous membranes and can be detected in a wound discharge. For comparison, we decided to examine the effect of lysozyme on microorganisms and lysis of the same bacterial cells in the presence of sodium dodecyl sulfate (SDS), which is part of IL-2-based drugs.

## EXPERIMENTAL SECTION

The following reagents were used: roncoleukin (0.25 mg/mL solution of purified interleukin-2 for intravenous and subcutaneous administration, Biotech, Russia); MES, Tris (“extra pure,” Amresco, USA); lyophilized hen egg lysozyme (95% purity, Sigma Aldrich, USA); NaOH (98% purity, AppliChem Panreac, Germany); CH_3_COOH (“AR grade,” Reachim, Russia); HCl (Germed, Germany); and a 10% water solution of SDS (BioRad, USA).

Microbial strains isolated from clinical specimens (urine, sputum, feces, wound discharge, etc.) were kindly provided by I.M. Sechenov First MSMU. The species of microorganisms were identified by direct protein profiling using MALDI-TOF mass spectrometry (FLEX series, Bruker Daltonic GmbH, Germany). A solid agar medium, 5% Colombia blood agar (Oxoid, UK), pH 7.3, was used for cultivation. The cell culture was grown at 35°C and 5% CO_2_ for 24 hours.

Strains from the museum collection of microorganisms (CM) of the Department of Microbiology at M.V. Lomonosov Moscow State University (referred to as MSU CM) were also used for the study. *Lactobacillus acidophilus *MSU CM 146, *Lactobacillus casei *MSU CM 153, and *Lactococcus lactis *MSU CM 165 were grown in a MRS liquid medium at 37 °C under anaerobic conditions [4]. *Clostridium butiricum *MSU CM 19 was grown in a medium of the following composition: 10 g/L glucose, 10 g/L peptone, 1 g/L K_2_HPO_4_, 5 g/L CaCO3, tap water; at 37 ° C under anaerobic conditions [5]. *Alcaligenes faecalis *MSU CM 82, *Bacillus megaterium *MSU CM 17, *Bacillus mycoides *MSU CM 31, *Bacillus cereus *MSU CM 9, *Pseudomonas aeruginosa *MSU CM 47, *Pseudomonas fluorescens *MSU CM 71, *Serratia marcescens* MSU CM 208, and *Staphylococcus aureus *MSU CM 144 were grown in a meat-peptone broth at 30 °C under aerobic conditions [6].

Lyophilized *Bifidobacterium bifidum *(Microgen, Russia) was used for the preparation of a suspension (10 mL of water per ampoule) at the initial stages of the study. Based on the analogy with the sample of lyophilized* L. plantarum *cells, it was assumed that the lyophilized bacterial sample differs little in the change of lysis rate from freshly grown cells [7].

*Thermus aquaticus *cells were graciously provided by A.A. Belogurov. Cells were grown according to the standard procedure for the culture at 75 °C under aerobic conditions [8].

Before measurements, all samples of bacterial cells were centrifuged at 3500 rpm for 4 min in a Minispin centrifuge (Eppendorf, Germany) then re-suspended in the buffer solution that was used for measuring the activity. The hen egg lysozyme solution was prepared immediately before the experiment using the same buffer as for activity measurement. A ready-to-use sample of IL-2 was used without additional treatment as a standard solution, and the ampoule was opened immediately before the experiment. Since the initial solution of IL-2 contained SDS (2.5 mg/mL), experiments on the effect of this component on background cell lysis were conducted. In order to determine the changes in absorption upon cell lysis, double-beam spectrophotometers UV-1800 or UV-1601PC (Shimadzu, Japan) were used. Measurements were performed in cells with an optical path length of 1 cm and a volume of 0.5 mL.

Bacteriolytic activity was determined turbidimetrically by a decrease in absorbance of cell suspension [7, 9] at a wavelength of 650 nm and a temperature of 37°C. A change in absorbance (A_650_) in the range of 5 to 20–30 s from the start of the reaction was used as the initial cell lysis rate. If background spontaneous lysis of cells took place in the absence of bacteriolytic factors, then its value was subtracted from the value of activity in the presence of bacteriolytic additives. In case of cell lysis in the presence of SDS, the value of the lysis rate in the presence of IL-2 was taken into account as a correction proportionally to the content of SDS in the sample. Cell suspension with an initial absorbance *A*650 = 0.4 was used for the determination of the cell lysis rate. The activity was measured in a 10 mM buffer solution of MES-Tris-CH_3_COOH at different pH values. As a relative value of activity, values of changes in the initial absorbance -dA/dt (AU/min) are presented, which (with the coefficients for corresponding cells) are proportional to the rate of change in the number of living cells or colony-forming units (-dCFU/dt), proportional to the changes in the lysis rate dΘ/dt (Θ = 0 if all cells remained intact, and Θ = 1 in case of 100% cell lysis) [7, 9].

## RESULTS AND DISCUSSION

The *Table* shows data on the effects of IL-2, lysozyme, and SDS on the cells of 37 strains of 34 different bacterial species. As one can see, 12 bacterial species are susceptible to lysis in the presence of lysozyme, 16 species are lysed in the presence of SDS, and only six species are sensitive to IL-2: *L. acidophilus*, *B. megaterium* (confirmed for two strains of the species)*, B. mycoides*,* B. cereus, S. marcescens, *and *Enterobacter aerogenes*. At the same time, lysozyme, IL-2, and SDS are active against *L. acidophilus *and *B. mycoides*. *B. megaterium*,* B. cereus, *and *S. marcescens *are susceptible to lysozyme and IL-2 but not SDS. *Ent. aerogenes *is only susceptible to IL-2. In general, the spectra of microorganisms sensitive to lysozyme and interleukin-2 are not identical, though they overlap. Apparently, the mechanisms of action differ starkly for lysozyme and IL-2.

**Fig. 1:**
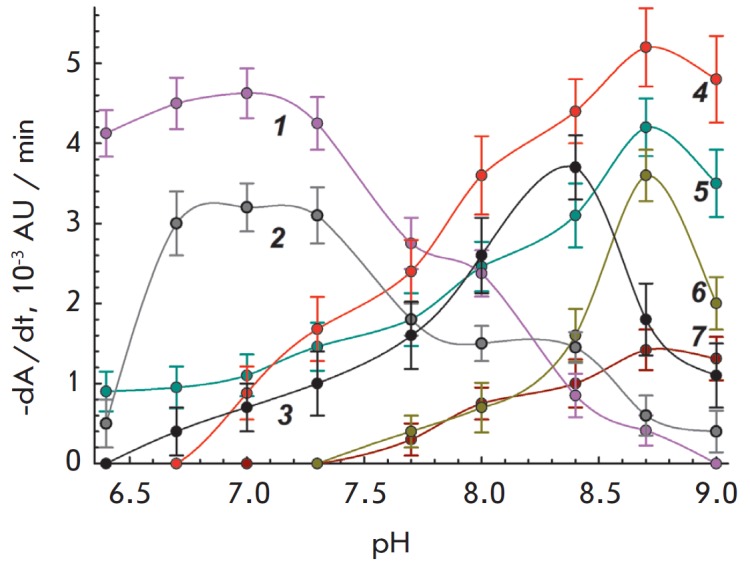
Dependence of cell lysis rate on pH in the presence of lysozyme. *1 *– *Streptococcus agalactiae*, lysozyme 5.0 μg/mL. *2 *– *Lactobacillus acidophilus *MSU CM 146, lysozyme 0.8 μg/mL. *3 *– *Serratia marcescens *MSU CM 208, lysozyme 0.2 μg/mL. *4 *– *Bacillus megaterium*, lysozyme 0.8 μg/mL. *5 *– *Pseudomonas aeruginosa*, lysozyme 0.2 μg/mL. *6 *– *Proteus vulgaris*, lysozyme 2 μg/mL. *7 *– *Staphylococcus haemolyticus*, lysozyme 0.4 μg/mL

The pH-dependence of the rate of cell lysis by lysozyme and IL-2 is presented in *Figs. 1 *and *2*. As can be seen, the values of pH-optimum activity for IL-2 and lysozyme against *B. megaterium *cells are identical and equal to 8.7. In the case of *L. acidophilus*, the pH-optima of lysozyme and IL-2 activity are also similar (6.5– 7.0 and 6.7–7.3). Activity optima for lysozyme and IL-2 are similar for *B. mycoides *and *B. cereus *(not presented on the graphs due to the similarity with the dependencies for *B. megaterium*). A similar shift in lysozyme and IL-2 activity optima depending on the substrate (species of bacteria) was also observed in the case of *E. coli* and *L. plantarum *[3].

**Fig. 2:**
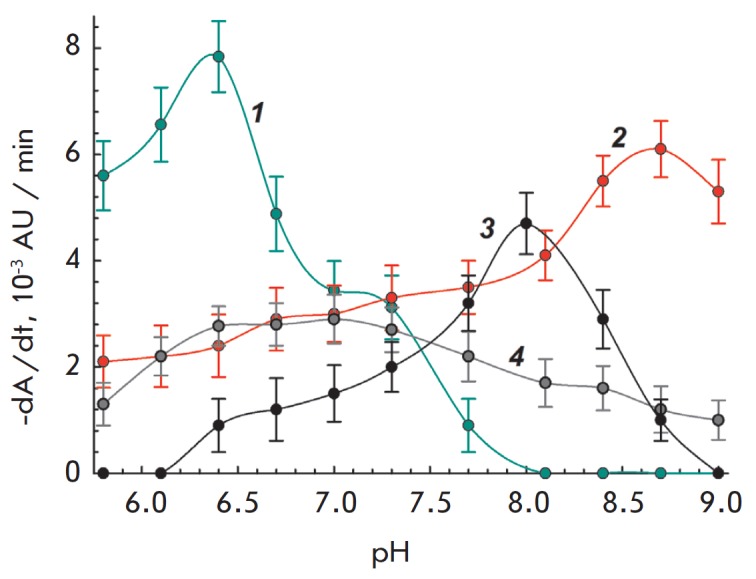
pH-dependence of cell lysis rate in the presence of interleukin-2. *1 *– *Enterobacter aerogenes*, interleukin-2 2.0 μg/mL. *2 *– *Bacillus megaterium*, interleukin-2 15 μg/mL. *3 *– *Serratia marcescens *MSU CM 208, interleukin- 2 30 μg/mL. *4 *– *Lactobacillus acidophilus *MSU CM 146, interleukin-2 5.0 μg/mL

**Fig. 3:**
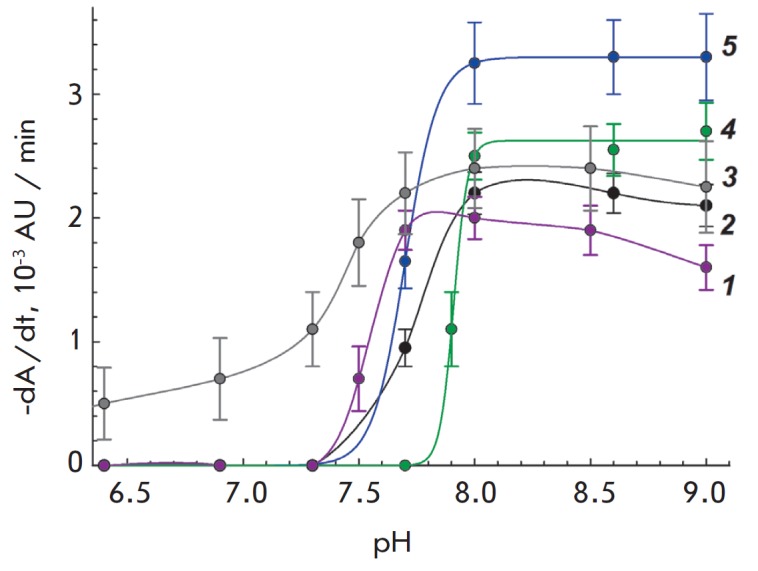
pH-dependence of cell lysis rate in the presence of SDS. *1 *– *Morganella morganii*, SDS 40 μg/mL.* 2 *– *Proteus vulgaris*, SDS 60 μg/mL. *3 *– *Lactobacillus acidophilus *MSU CM 146, SDS 50 μg/mL. *4 *– *Pseudomonas putida, *SDS 0.2 mg/mL. *5 *– *Stenotrophomonas maltophilia, *SDS 0.15 mg/mL

*Fig. 3* shows the pH-dependence of the cell lysis rate in the presence of SDS. The graph presents data for only five of the 16 microorganisms sensitive to SDS. For the other 11 microorganisms, pH-dependences of the cell lysis rate in the presence of SDS are similar. As it can be seen, SDS acts best on cells in an alkaline medium, which is inherent to various microorganisms. SDS is active at pH higher than 7.3–8.0. It is possible that such a tendency of pH-dependence is somehow connected to the range of pK values of the phosphate groups of cell membrane phospholipids. It is also possible that the components of the buffer solution (for example, Tris) can influence the nature of the pH-dependence. Identification of the exact molecular reason for such pH-dependency of the SDS action is beyond the scope of our study.

IL-2 acts on individual members of the Gram-negative family Enterobacteriaceae, including *Ent. aerogenes* and *S. marcescens*, as shown in our work, and, as previously established, on *E. coli *[1-3]. IL-2 is active against such Gram-positive members of the family Lactobacillaceae as *L. acidophilus *(current paper) and* L. plantarum *[3]. It was also found that IL-2 acts on *B. megaterium*, *B. mycoides, *and *B. cereus*, Gram-positive spore-forming bacilli of the Bacillaceae family, which differ in cell wall structure and composition from the bacteria of the Enterobacteriaceae and Lactobacillaceae families. It can be assumed that the cell walls of* E. coli*, *Ent. aerogenes*, *S. marcescens*, *L. plantarum*, *L. acidophilus*, *B. mycoides*, *B. megaterium *and *B. cereus* have some similar structures. Indeed, structures containing diaminopimelic acid have been detected in the cell wall of *B. megaterium*, *B. cereus *and *L. plantarum* [10-13], which are not typical for many Gram-positive microorganisms but quite common among representatives of the family Enterobacteriaceae [13, 14]. The cell wall of *L. acidophilus *is believed not to contain significant amounts of diaminopimelic acid [15]. However, we can assume by analogy with *L. plantarum *that diaminopimelic acid may comprise the cell wall of certain strains of *L. acidophilus*. We have not found any publications demonstrating accurate data on the presence and quantity of diaminopimelic acid in *B. mycoides*, but we can assume that the structure of the cell wall of this bacterium, *B. megaterium *and *B. cereus,* can be partially similar. Apparently, similarity in susceptibility to IL-2 of such unrelated microorganisms can be explained by the presence of common structures containing diaminopimelic acid. We have previously shown that IL-2 has no effect on *B. subtilis *cells [1, 2], which also belong to the family Bacillaceae. However, some data have been published according to which, in contrast to many other members of this family, *B. subtilis* contains diaminopimelic acid, which is presented in amidated form [16]. Thus, the resistance of *B. subtilis* to IL-2 actually confirms our hypothesis. In general, it is too early to draw accurate conclusions at this stage of the study about what types of microorganisms are sensitive to IL-2. Moreover, sensitivity to bacteriolytic agents can vary depending on the presence and composition of the capsule in bacteria, as well as vary even among different strains of the same species [17]. It should be noted that there is ongoing debate on the mechanisms of lysozyme action, which has been studied for a long time, against various microorganisms. There are reasons to believe that lysozyme can act not only on bacterial cells as an enzyme, but also as a cationic antibacterial protein [18]. As a result of our work, we established the spectrum of microorganisms sensitive to interleukin-2, which will help further study the molecular mechanisms of susceptibility or immunity of microorganisms to this bacteriolytic factor.

**Table:** Lysis of bacteria in the presence of interleukin-2, lysozyme and sodium dodecyl sulfate (SDS)

№	Microorganism	Cell lysis rate in the presence of an additive		
		lysozyme	interleukin-2	SDS
1	*Acinetobacter baumannii*	0	0	0
2	*Alcaligenes faecalis MSU CM 82*	3.2/2.0/6.4	0	1.1/100/8.0
3	*Bacillus megaterium*	5.2/0.8/8.7	6.1/15/8.7	0
4	*Bacillus megaterium MSU CM 17*	2.2/2.0/8.5	2.6/30/8.5	0
5	*Bacillus mycoides MSU CM 31*	4.5/4.0/8.0	3.6/10/8.0	0.7/100/8.0
6	*Bacillus cereus MSU CM 9*	4.5/4.0/8.5	0.9/30/8.5	0
7	*Bifidobacterium bifidum*	0	0	0
8	*Citrobacter braakii*	0	0	0
9	*Clostridium butiricum MSU CM 19*	0	0	2.5/400/8.0
10	*Corynebacterium amycolatum*	0	0	0
11	*Enterobacter aerogenes*	0	7.8/2.0/6.4	0
12	*Enterobacter cloacae*	0	0	0.9/200/8.0
13	*Enterococcus faecalis*	0	0	1.9/50/8.0
14	*Klebsiella pneumoniae*	0	0	0
15	*Lactobacillus acidophilus MSU CM 146*	3.2/0.8/7.0	2.9/5.0/7.0	2.4/50/8.0
16	*Lactobacillus casei MSU CM 153*	0	0	0
17	*Lactococcus lactis MSU CM 165*	0	0	0
18	*Morganella morganii*	0	0	2.0/40/8.0
19	*Neisseria perflava*	0	0	0
20	*Proteus mirabilis*	0	0	2.9/50/8.0
21	*Proteus vulgaris*	3.6/2.0/8.7	0	2.2/60/8.0
22	*Pseudomonas aeruginosa*	4.2/0.2/8.7	0	5.8/50/8.0
23	*Pseudomonas aeruginosa MSU CM 47*	7.3/0.4/7.7	0	1.1/100/8.0
24	*Pseudomonas fluorescens MSU CM 71*	3.5/0.5/8.4	0	0
25	*Pseudomonas putida*	0	0	2.5/200/8.0
26	*Rothia mucilaginosa*	0	0	0
27	*Serratia marcescens MSU CM 208*	3.7/0.2/8.4	4.7/30/8.0	0
28	*Staphylococcus aureus*	0	0	6.2/50/8.0
29	*Staphylococcus aureus MSU CM 144*	1.6/1.0/7.7	0	0
30	*Staphylococcus capitis*	0	0	0
31	*Staphylococcus epidermidis*	0	0	0
32	*Staphylococcus haemolyticus*	1.4/0.4/8.7	0	4.9/20/8.0
33	*Staphylococcus lugdunensis*	0	0	0
34	*Stenotrophomonas maltophilia*	0	0	3.3/150/8.0
35	*Streptococcus agalactiae*	4.6/5.0/7.0	0	4.1/50/8.0
36	*Streptococcus pyogenes*	0	0	0
37	*Thermus aquaticus*	0	0	3.6/125/8

*Note. *Values of the lysis rate are presented in the form X/Y/Z, wherein X is the lysis rate, AU, 10^-3^ × min^-1^, Y is the concentration of an additive, μg × mL^-1^, and Z is pH of the medium at which the measurements were made. Values of pH-optimum are presented for lysozyme and interleukin-2: all rate values for SDS were obtained at pH 8.0. Zeroes indicate that no absorbance change was obtained for 3 min at concentrations of up to 5 μg/mL, 50 μg/mL and 0.5 mg/mL for lysozyme, interleukin-2, and SDS, respectively.

## Abbreviations

CFU: the number of colony forming units
SDS: sodium dodecyl sulfate
